# Toxin–Host Interaction of *Clostridium* Toxins

**DOI:** 10.3390/toxins18020067

**Published:** 2026-01-27

**Authors:** Jonatan Dorca-Arévalo

**Affiliations:** 1 Department of Pathology and Experimental Therapeutics, Faculty of Medicine and Health Sciences-Campus Bellvitge, University of Barcelona, 08907 Barcelona, Spain; jondorca@ub.edu; 2 Laboratory of Molecular and Cellular Neurobiology, Bellvitge Biomedical Research Institute (IDIBELL), 08907 L’Hospitalet de Llobregat, Spain; 3 Institute of Neuroscience, Bellvitge Health Sciences Campus, University of Barcelona, Carrer de la Feixa Llarga, s/n, 08907 L’Hospitalet de Llobregat, Spain

Clostridial toxins, involved in a wide range of serious diseases, affect humans and animals. Among the best known are the neurotoxins produced by *Clostridium botulinum* and *Clostridium tetani*, which are considered to be one of the most potent bacterial toxins known, with botulinum toxin being the most potent biological toxin identified to date. In addition, many clostridial toxins cause gastrointestinal disorders that can lead to severe health complications and, in some cases, death. In this Special Issue (SI), “Toxin–Host Interaction of *Clostridium* Toxins”, we present recent research on toxins produced by *Clostridioides difficile*, the most significant species due to its clinical impact and high prevalence, particularly in hospital settings, and *Clostridium perfringens*, which causes severe infections such as gas gangrene, a rapidly fatal condition, as well as enterotoxemia.

The toxins produced by *Clostridioides difficile*, TcdA and TcdB, possess glucosyltransferase activity and cause severe symptoms ranging from diarrhea to pseudomembranous colitis. Both toxins are internalized into host cells via receptor-mediated endocytosis. Their enzyme domains glucosylate small GTPases, thereby disrupting actin cytoskeleton regulation. Interestingly, pharmacological inhibition of Hsp70 using the inhibitor VER-155008, as well as the antiemetic drug domperidone, was shown to protect cells from TcdB intoxication. Domperidone prevented the membrane translocation of TcdB’s glucosyltransferase domain into the cytosol and also conferred protection against TcdA and the binary CDT toxin produced by hypervirulent *C. difficile* strains [1]. These findings identify Hsp70 as a promising therapeutic target against *Clostridioides difficile*.

Regarding *Clostridium perfringens*, it is a spore-forming, Gram-positive anaerobic pathogen that causes various disorders in humans and animals. Recently, a multidrug-resistant strain (IRMC2505A) was isolated from the fecal sample of a patient clinically suspected of a gastrointestinal infection, who had a recent history of antibiotic exposure and diarrhea. Nineteen antimicrobial resistance genes and virulence factors were identified in the IRMC2505A strain through 16S rRNA sequencing, followed by whole-genome analysis and bioinformatics mapping against the CARD and VFDB databases. This is the first whole-genome report of this strain from Saudi Arabia and highlights the need to understand its resistance and virulence profiles to guide control strategies [2].

The most lethal toxin produced by *Clostridium perfringens* is the epsilon toxin (ETX). This toxin, produced by types B and D, causes generalized edema and is capable of crossing the blood–brain barrier, resulting in neurotoxicity that can lead to animal death. Interestingly, ETX has recently been linked to a potential association with multiple sclerosis in humans, although further studies are required to confirm this relationship. While ruminants are the natural hosts for *C. perfringens* types B and D and, consequently, for ETX-mediated neurotoxicity, laboratory mouse models have been developed that exhibit similar toxic effects, allowing experimental investigation of the toxin’s mechanisms. ETX is classified as a member of the pore-forming toxin (PFT) family due to its ability to form pores in the plasma membrane of sensitive cell types such as MDCK, 1G11, FRT, and MOLT4 cells. Despite this classification, the specific molecular changes induced in affected cells that ultimately lead to cell death remain poorly understood. To address this knowledge gap, a recent study employed quantitative proteomics and phosphoproteomics, utilizing TMT labeling and LC–MS/MS, to systematically analyze changes in protein expression and phosphorylation in MDCK cells exposed to ETX. This approach revealed a large number of differentially expressed and phosphorylated proteins, many of which were enriched in pathways related to RNA processing and the spliceosome, implicating these processes as central components of ETX-induced cellular toxicity. Functional validation experiments using pharmacological inhibitors and RNA interference further demonstrated that key proteins, including SRSF1, SF3B2, THOC2, and SRPK1, which are involved in spliceosome-related pathways, play a critical role in mediating ETX-induced cell death. These findings suggest that disruption of mRNA splicing is a major mechanism underlying ETX toxicity and provide a foundation for future research aimed at identifying therapeutic targets to mitigate the effects of this potent neurotoxin [3].

The Myelin and Lymphocyte (MAL) protein is the most widely accepted putative receptor and is primarily expressed in myelin and in the plasma membrane of T-lymphocytes. In this context, an interesting study investigating the cytotoxic effects of ETX on primary human lymphocytes demonstrated that ETX preferentially binds to and kills these cells in a time- and dose-dependent manner. Using flow cytometry, the authors quantified ETX binding and cytotoxicity across lymphocyte subsets (CD4^+^, CD8^+^, and CD19^+^), finding the highest binding and killing in CD4^+^ cells. To determine whether receptor expression accounted for this cell-type preference, the study measured MAL gene expression by RT-qPCR, showing that CD4^+^ lymphocytes express higher levels of MAL. These results indicate that primary human lymphocytes are susceptible to ETX and support the hypothesis that MAL is a key receptor mediating toxin binding. The findings suggest that ETX may influence human immune responses and potentially implicate immune cells in conditions like multiple sclerosis [4]. These results align with the results obtained by Dorca-Arévalo’s laboratory using MOLT4 cells. In addition, the generation of extracellular vesicles (EVs) for the affected cells is induced by ETX, and this action is dependent on MAL-GFP expression in the plasma membrane, as demonstrated by the HeLa cell line. Moreover, in those EVs, the presence of ETX and MAL was detected, suggesting that host cells may shed EVs in response to pore formation, as other PFTs can do [5]. This finding provides a new tool to study the mechanism of ETX action and offers insight into how ETX interacts with its receptor.

Vaccines play a critical role in preventing *Clostridium* infections, and there is currently intense research focused on developing new, more efficient strategies to improve safety, immunogenicity, and coverage. In line with these efforts, a recent review traces the evolution of vaccine design from early live attenuated and inactivated vaccines to modern approaches, including recombinant protein and subunit vaccines targeting bacterial toxins. It highlights advances such as genetically detoxified proteins, mRNA platforms, and other novel technologies, emphasizing how a deeper understanding of pathogen–host interactions can guide the development of safer and more effective toxin-based vaccines [6]. Building on this, a recent study developed a dissolving microneedle (dMN) vaccine for *Clostridium perfringens* epsilon toxin (ETX) by formulating recombinant ETX protein with poly(lactic-co-glycolic acid) (PLGA) nanoparticles modified with dimethyldioctadecylammonium bromide (DDAB) to enhance antigen adsorption and immune response. PLGA nanoparticles were prepared and integrated into a fish gelatin microneedle matrix that could successfully penetrate skin. In vivo immunization via the dMN patch and subcutaneous injections revealed that both routes induced similar antibody titers, but the dMN vaccine provided superior protection in neutralization assays against lethal ETX challenge.

In vivo neutralization experiments showed 100% survival in the dMN group challenged with 100 × LD_50_ ETX, while the aluminum-adjuvant group showed lower survival, demonstrating the improved protective efficacy of the PLGA-based microneedle vaccine [7].

These promising experimental approaches complement broader research efforts, as highlighted in a recent review summarizing the epidemiology, toxin biology, and vaccine development strategies for *Clostridium perfringens*. The review provides a comprehensive overview of current knowledge on the pathogenesis of *C. perfringens* toxins, their molecular mechanisms, and ongoing attempts to develop effective immunization strategies, emphasizing both traditional toxoid vaccines and novel platforms such as recombinant and nanoparticle-based vaccines [8]. Importantly, the review does not identify a single superior vaccine; instead highlights the ongoing need for research to develop safer and more effective immunization approaches.
